# Private school canteens: an analysis of the economic and financial aspects of the traditional and the healthy models

**DOI:** 10.1186/s12889-023-16965-1

**Published:** 2023-10-25

**Authors:** Luisa Arantes Vilela, Bruna Vieira de Lima Costa, Mariana Zogbi Jardim, Luiza Delazari Borges, Ariene Silva do Carmo, Monique Louise Cassimiro Inácio, Larissa Loures Mendes

**Affiliations:** 1 Department of Nutrition, Federal University of Minas Gerais, 190 Professor Alfredo Balena avenue, Santa Efigenia, Belo Horizonte, MG 30130-090 Brazil; 2 Department of Pediatrics, Federal University of Minas Gerais, 190 Professor Alfredo Balena avenue, Santa Efigenia, Belo Horizonte, MG 30130-090 Brazil; 3 Researcher, Group of Studies, Research and Practices in Food Environment and Health (GEPPAAS), Federal University of Minas Gerais, 190 Professor Alfredo Balena avenue, Santa Efigenia, Belo Horizonte, MG 30130-090 Brazil; 4 School of Nutrition, Federal University of Ouro Preto, Campus, s/n, Morro do Cruzeiro, Ouro Preto, MG 35400-000 Brazil

**Keywords:** School canteens, School meals, School food environment, Economic viability

## Abstract

**Background:**

One of the reasons for the more prominent resistance of canteen managers to implementing healthy canteens is based on the belief in the economic infeasibility of these models. The research aimed to verify the economic and financial viability of traditional and healthy models of school canteens in a Brazilian metropolis.

**Methods:**

The case study was carried out with 36 companies in the school canteen sector in a Brazilian metropolis. The classification of items sold in canteens considered the extent and purpose of food processing according to the NOVA Classification. The characterization and definition of traditional canteens and healthy canteens were proposed considering the amount of in natura or minimally processed foods and culinary preparations without the presence of ultra-processed foods; the percentage of ultra-processed foods or processed foods or culinary preparations with the presence of ultra-processed foods; and the existence of prohibited foods. The economic and financial analysis was carried out mainly through the evaluation of profitability. Data were collected through an electronic self-administered questionnaire sent to canteen managers. The Mann-Whitney test was used to compare medians and the Chi-Square/Fisher’s Exact Test to compare proportions.

**Results:**

The study included six companies, responsible for 36 canteen units in private schools, 30 classified in the traditional model (83.3%), and six in the healthy model (16.7%). The median percentage of natural, minimally processed foods and commercialized culinary preparations was higher among the healthy model canteens (87.9% vs. 60.0%, p < 0.001). While the median percentage of ultra-processed, processed, or preparations with the presence of ultra-processed (40.0% vs. 12.1%, p < 0.001) and prohibited foods (10.0% vs. 0%, p < 0.001) sold was higher in the traditional model canteens. The results indicated that the profitability in the healthy canteens was higher (p < 0.001) than in the traditional ones.

**Conclusions:**

Healthy school canteens showed better financial and economic results compared to traditional canteens with emphasis on greater profitability and a shorter recovery time of the initial investment.

## Background

In Brazil, the sale of food in schools differs between public and private education networks in terms of the contract, the form of administration of the canteens, and the presence of the National School Feeding Program (PNAE in the Portuguese acronym) [1]. The canteen is understood to be an establishment located inside the school that aims to offer food services to the school community for a fee [2].

In private schools, the main option for access to food by students is the canteen. Data from the National School Health Survey - PENSE (2019) revealed the presence of canteens in three out of ten public schools and nine out of ten private schools [3]. These canteens can be managed by the school itself or by an outsourced company [1]. Studies show that the presence of canteens in private schools is associated with greater availability and consumption of unhealthy foods (ultra-processed foods and beverages - UPF) by students [4–9], which characterizes these spaces as a more obesogenic environment when compared to public schools [4, 5].

In addition, positive associations are observed between the consumption of unhealthy snacks sold in school cafeterias and students’ overweight/obesity [9–12]. On the other hand, regulations and interventions that reduce the availability of UPFs and increase the supply of in nature and minimally processed foods in canteens have shown positive results concerning improvements in food consumption, nutritional profile, and adherence to a healthy diet by the student population [13–17].

In Brazil, a healthy diet based on in nature and minimally processed foods has a higher cost trend. However, due to the low cost of grains (rice and beans), healthy diets are still cheaper than those consisting of ultra-processed foods [18]. From this angle, Brazilian outsourced companies, those specialized in school lunches, which sell mostly in nature and minimally processed foods and beverages, have shown to be economically viable [1, 19, 20]. A project entitled “The School Promoting Healthy Eating Habits”, carried out by the Observatory of Food Security and Nutrition Policies at the University of Brasilia, carried out a survey that showed that 66.7% of school canteens that promoted and offered healthy snacks had an increase in profit between 30.0 and 50.0%, adding value to your business [21].

Despite this, there are some limitations to the provision of healthy foods and beverages in Brazilian schools, such as the low coverage of regulations in private schools, the lack of a national law that prohibits or restricts the sale of UPF in the school environment [6], inefficient supervision [22–26] and resistance to adherence and compliance with legal provisions by private schools [1]. In addition, it is common for canteen owners to report that the sale of healthy foods is low, generating little profit, which is, therefore, a limiting factor in the implementation of healthy canteens [22], in addition to restrictions on workers’ time to produce healthier meals, since there are reports of a low number of employees in the canteens considering the number of students in the school [27].

Some systematic reviews have also shown that concerns on the part of school principals or owners of food services on the loss of profitability, revenue and/or commercial viability constitute one of the main barriers to implementing healthy canteens [28, 29]. Thus, considering that one of the barriers identified for the implementation of healthy canteens is the greatest resistance on the part of canteen managers due to the belief about the economic unfeasibility of healthy models, studies that assess the economic viability of school canteens can contribute with evidences that will be important to in raising awareness and training canteen owners. Also, it contributes to the promotion of a healthier school food environment. However, there are still few studies that assess the economic viability of healthy canteens.

### Aim

The objective of this study was to evaluate the economic and financial aspects of different models of companies in the school canteen sector in a Brazilian metropolis.

## Methods

### Design and setting of the study

This is a case study that evaluated the economic and financial aspects of private school canteens carried out in the city of Belo Horizonte, Brazil. Belo Horizonte is the sixth most populous city in the country with approximately 2,375,151 inhabitants [30]. In 2010, the Municipal Human Development Index (IDHM in the Portuguese acronym), which considers the dimensions of longevity, education, and income, was 0.810, ranking the city in 20th position among Brazilian municipalities [30].

### Participants

To participate in the study, the companies managing the school canteens had to have a service contract with private schools, which offered Elementary School and/or High School. Schools that only had Early Childhood Education were excluded since in these cases it is more common for the school itself to administer the canteen (self-management) and/or for the parents to send the child a snack from home.

According to data from the Minas Gerais State Department of Education (SEE/MG in the Portuguese acronym), in 2019, Belo Horizonte had 902 schools in the private network, 499 of which were exclusively for kindergarten. In this way, 403 schools were contacted by telephone to obtain information on how the canteen is managed (self-management or outsourced) and on the responsible manager. Of these, 84 had an outsourced canteen, 174 did not have a canteen or canteen with a third-party service (they were self-managed), 23 reported not having the authorization to provide data, and 122 did not answer the phone, even after two attempts in days and periods (morning and afternoon) alternated. Ethical approval for the study was granted by the Ethics in Research and Human Beings Committee of the Federal University of Minas Gerais (CAAE: 38003220.4.0000.5149; Opinion number: 4.454.467). Informed consent was obtained from all managers participants included in the study.

### Measures

The team responsible for data collection was composed of graduate students and researchers from a Research Group, who were duly trained. In the training, a pattern of approach was defined, by telephone or e-mail, and recording the answers. Most schools that had outsourced canteens provided the contact of the canteen manager who was later invited to participate in the research. Of the 84 schools with an outsourced canteen, 37 companies and managers were identified. All companies were contacted and 6 managers agreed to participate in the research, representing 42.9% (n = 36) of private schools with an outsourced canteen. Thus, the sample of school canteens was characterized by not being probabilistic, considering that random selection methods were not used.

Data collection took place between March and July 2021 and consisted of electronically sending a questionnaire to be self-completed by canteen managers. The questionnaire was sent to all managers of eligible companies and was prepared by the researchers of the study and reviewed by UFMG Consultoria Júnior (UCJ in the Portuguese acronym), a business management consulting company composed of students from the courses of Administration, Economic Sciences, Accounting Sciences, International Economic Relations, and Controllership and Finance at the Federal University of Minas Gerais (UFMG in the Portuguese acronym). The information investigated referred to the identification of the outsourced company, the linked school, number of canteens and students served, menu of commercialized products, product offering, number of employees, investment value and recovery time, profit margin of the most sold, monthly gross revenue, monthly expenses, company profitability, manager’s perceptions about the identification of healthy canteens, implementation feasibility and attempt to include healthy foods. It is noteworthy that all the information collected referred to the year 2019, before the beginning of the COVID-19 pandemic in Brazil. Caused by the SARS-CoV-2 betacoronavirus, COVID-19 was considered a public health emergency of international importance, forcing several countries to adopt social distancing measures, in addition to travel and street movement restrictions [31, 32]. Social distancing measures have involved school closures in approximately 137 countries [33]. In Brazil, it has been estimated that 189,707,136 students were affected by the closure of schools in the year 2020 [34]. Consequently, the entire canteen sector came to a halt considering the sanitary measures imposed to reduce the transmission of SARS-CoV-2.

#### Classification of foods sold in canteens

The classification of items sold in the canteens considered the extent and purpose of food processing, according to the NOVA Classification [35, 36], present in the Food Guide for the Brazilian Population [37], which are classified as in nature or minimally processed, processed, ultra-processed, and culinary ingredients. The Food Guide presents the following golden rule for healthy eating: always prefer natural or minimally processed foods and culinary preparations to ultra-processed foods. These culinary preparations would be based on in nature or minimally processed foods and may include culinary ingredients and, eventually, processed foods [37].

Considering the diversity of culinary preparations existing in school canteens, two food groups were created: (1) in nature, minimally processed foods and culinary preparations without the presence of ultra-processed foods; (2) processed foods, ultra-processed foods and culinary preparations with the presence of ultra-processed foods. It is noteworthy that for the classification of “culinary preparations with the presence of ultra-processed foods”, the presence of at least one ultra-processed food was considered as an ingredient in the preparation as mentioned in the menu description.

#### Healthy canteen and traditional canteen

The characterization and definition of which establishments would be considered traditional canteens and healthy canteens was based on articles 21 and 22 of Resolution nº 6 of May 8, 2020, of the PNAE [38] at least 75.0% in nature or minimally processed foods; (2) no more than 20.0% processed and ultra-processed foods; (3) the prohibition of certain ultra-processed foods and beverages, such as soft drinks and artificial refreshments, beverages or concentrates based on guarana or currant syrup, ready-to-drink teas and other similar beverages, cereals with additives or sweetened, candies and the like, confectionery, candy, chocolate bars and granules, cookies or filled cookies, cake with icing or filling, cereal bars with additives or sweetened, edible ice cream, gelatin, seasonings with monosodium glutamate or sodium salts, mayonnaise, and powdered or reconstituted foods.

In addition, the study by Rodrigues (2019) [39] was also used as a reference, as guidelines were established for the certification of healthy canteens, such as (1) expanding the supply of fresh and minimally processed foods; (2) limiting the supply of processed foods (reduction to 50.0% and then to 30.0%); (3) restrict the sale of ultra-processed foods (reduction to 40.0% and then to 20.0%); (4) prohibit the sale of candies, lollipops, chewing gum, stuffed cookies, soft drinks, artificial or sweetened juices, fried foods (such as rissoles, pastries, and drumsticks), mayonnaise, snacks with sausages, packaged snacks, industrialized popcorn, and others ultra-processed foods high in sodium, fat, and sugar. Table 1 presents the classification of canteens in the traditional and healthy models.

**Table 1:** Classification of school canteens proposed by the present authors and adapted from other criteria in the literature [36, 37]

	Traditional canteen	Healthy canteen
**In nature or minimally processed foods and culinary preparations without the presence of ultra-processed**	< 80,0%	≥ 80,0%
**Ultra-processed foods or processed foods or culinary preparations with the presence of ultra-processed foods**	≥ 20.0%	< 20.0%
**Prohibited foods:** candies, confectionery, bonbon, chocolate bars and sprinkles, cake with icing or filling, cereal bar with additives or sweetened, edible ice cream, gelatin, lollipops, chewing gum, stuffed cookies, soft drinks, artificial juices or refreshments or sweeteners, beverages or concentrates based on guarana or currant syrup, ready-to-drink teas and other similar beverages, cereals with additives or sweeteners, fried snacks, mayonnaise, snacks with sausages, packaged snacks, industrialized popcorn, seasonings with monosodium glutamate or sodium salts	Presence of one or more prohibited foods	No prohibited food

In this way, the analysis of the products sold in the canteens was carried out through the evaluation of the menu provided by the canteen manager. For this analysis, the number of items offered for sale was considered; the amount of in nature or minimally processed foods and culinary preparations without the presence of ultra-processed foods; the amount of processed food, ultra-processed foods, and culinary preparations that contain ultra-processed foods; and the amount of prohibited foods.

#### Economic and financial aspects of canteens

The analysis of the financial statements can be divided into economic analysis, which includes the interpretation of the results generated by the company, and financial analysis, which includes the financial availability of the company, its degree of liquidity, and its ability to pay. Thus, there are several techniques for the economic-financial analysis of a company, which are essential tools for controlling the organization’s financial situation and for decision-making by managers [40, 41]. In the present study, it was proposed to use the analysis through the evaluation of profitability, which is all that is left net of the gross revenue of the establishment after all expenses have been paid [40]. This indicator is calculated from the income statement for the year (DRE in the Portuguese acronym), a financial statement that presents the company’s economic results determining the profit or loss in a period [40]. Dornelas (2012) [42] defines the DRE as an ordered and summarized classification of the company’s income and expenses in a given period. Taxes, allowances, and refunds granted are subtracted from total revenue, resulting in net revenue; from net revenue, the costs arising from products sold, products manufactured, or services rendered are deducted, to arrive at a gross profit; subsequently, operating expenses are subtracted from gross profit; and finally, the income tax is calculated, accounting at the end of the sum of profits and losses.

Information on the initial investment value was requested from the participants and in the present study, it refers to that fixed, which corresponds to the equipment, utensils, and furniture necessary for the operation of the enterprise, disregarding the value for the working capital [43]. To obtain the net profit value, the reported value was multiplied as a percentage of profitability over the gross revenue value, reported in reais. The calculation of the estimated total expenses of the canteens was performed by subtracting the net income from the gross revenue. The stratification of costs was classified into fixed costs which refer to those that are independent of the product sold and variable costs those that are directly related to the amount of product sold, as described by Kimura (2003) [44]. In this sense, expenses with infrastructure, employees, and financial services (accounting, sales management system, bank fees, fire insurance) were denominated as fixed costs, and as variable costs, expenses with foodstuffs, cleaning products, and extra expenses (office supplies, equipment maintenance, extermination, grease and water tank cleaning, or other expenses not mentioned). In addition to these data, the number of schools served by each company, the average number of people served, and the average number of employees that make up the company were also evaluated.

### Statistical analyses

The descriptive analysis included the calculation of frequency distributions and measures of central tendency and dispersion. The Shapiro-Wilk normality test was applied. Median and interquartile ranges (25th and 75th percentiles) were calculated for non-parametric quantitative variables, and absolute and relative frequencies were calculated for categorical variables.

The Mann-Whitney Test was used to compare medians and the Chi-Square/Fisher Exact Test to compare proportions. In situations where statistical significance was found in the Chi-Square Test, the 2 × 2 analysis was used to identify possible differences. In this analysis, the Bonferroni correction was used, which changes the level of significance (p), to avoid type I errors derived from multiple comparisons [45].

All information obtained was recorded in a computerized database, prepared for this purpose with the aid of Excel 11.0 software. Data analyzes were performed using the statistical software Statistical Package for the Social Sciences (SPSS), version 19.0 [46]. The significance level adopted in all analyzes was 5.0%.

## Results

The study was carried out on a total of six outsourced companies that were responsible for 36 canteen units. According to the methodology proposed to classify the canteens of private schools, 83.3% (n = 30) followed the traditional food marketing model, and 16.7% (n = 6) the healthy food marketing model.

Table 2 presents the description of the items and types of food sold according to the canteen models. The average of items sold was higher in traditional canteens than in healthy ones (40 vs. 33, p = 0.003). The average percentage of in nature, minimally processed and preparations without the presence of ultra-processed foods was higher among the healthy model canteens (87.9% vs. 60.0%, p < 0.001). While the average percentage of ultra-processed, processed, or culinary preparations with the presence of ultra-processed (40.0% vs. 12.1%, p < 0.001) and prohibited foods (10 vs. 0, p < 0.001) sold was higher in the traditional model canteens when compared to the healthy model canteens (Table 2). Savory foods (96.7%, n = 29), followed by natural juice (80.0%, n = 24), were the most commercialized foods in the traditional model canteens. In the canteens of the healthy model, cheese bread (100.0%, n = 6) was highlighted in sales (data not shown).

**Table 2:** Description of the items and types of food sold in the canteens in the traditional and healthy models

	Traditional model (n = 30)	Healthy model (n = 6)	p value*
**Number of items sold (n)**	40 (40–40)	33 (31–33)	**0,003**
**In nature or minimally processed foods and preparations without the presence of ultra-processed (%)**	60,0 (56,6–60,0)	87,9 (87,9–90,3)	**< 0,001**
**Ultra-processed foods or processed foods or culinary preparations with the presence of ultra-processed foods (%)**	40,0 (40,0–43,4)	12,1 (9,7–12,1)	**< 0,001**
**Prohibited foods (n)**	10 (10–10)	0 (0–0)	**< 0,001**

Note: values presented as median and interquartile range (25th and 75th percentiles)

*Mann-Whitney Test

Statistically significant differences were observed between the two canteen models for the average number of students served (p < 0.001), the time to recover the investment (p = 0.005), profit margin of the best-selling product (p < 0.001), costs fixed with infrastructure (p < 0.001), with financial services (p < 0.001), with disposables (p < 0.001), with cleaning products (p < 0.001) and with extra expenses (p < 0.001) (Table 3). We proceeded with the Bonferroni correction to verify in which pair or pairs of comparison there is a statistical difference (data not shown). After the Bonferroni correction, it was found that there was no difference in spending on employees according to the canteen model (Table 3).

**Table 3:** Characterization of canteens in the traditional and healthy models according to economic and financial aspects

	Traditional model (n = 30)	Healthy model (n = 6)	p value*
**Average number of people served in the canteen**			
0 to 150 150 to 300 300 to 500 > 500	0 (0,0%) 0 (0,0%) 0 (0,0%) 30 (100,0%)	0 (0,0%) 0 (0,0%) 6 (100,0%) 0 (0,0%)	**< 0,001**
**Average number of employees**			
0 to 5 employees 6 to 10 employees 11 to 15 employees 16 or more employees	0 (0,0%) 2 (6,7%) 0 (0,0%) 28 (93,3%)	0 (0,0%) 2 (33,3%) 0 (0,0%) 4 (66,7%)	0,058
**Average initial investment value (BRL)**	30.000	30.000	1,000
	(30.000–30.000)	(30.000–30.000)	
**Time to recoup the investment (years)**			
< 1 1 to 3 3 to 5 5 to 10 > 10 Haven’t recoup	0 (0%) 29 (96,7%) 1 (3,3%) 0 (0,0%) 0 (0,0%) 0 (0,0%)	2 (33,3%) 4 (66,7%) 0 (0,0%) 0 (0,0%) 0 (0,0%) 0 (0,0%)	**0,005**
**Profit margin of the best-selling product (%)**			
0 to 33 33 to 66 66 to 100 > 100	5 (16,7%) 1 (3,3%) 1 (3,3%) 23 (76,7%)	0 (0,0%) 6 (100,0%) 0 (0,0%) 0 (0,0%)	**< 0,001**
**Gross Revenue (BRL)**	200.000	130.000	0,134
	(100.000-200.000)	(100.000-130.000)	
**Total Expenses (BRL)**	180.000	97.500	0,100
	(94.000-180.000)	(82.000-97.500)	
**Fixed Costs (BRL)**			
*Infrastructure*			
0 to 2.500 2.500 to 5.000 5.000 to 7.500 > 7.500	28 (93,3%) 1 (3,3%) 0 (0,0%) 1 (3,3%)	(0,0%) 4 (66,7%) 2 (33,3%) 0 (0,0%)	**< 0,001**
*Employee spending*			
0 to 5.000 5.000 to 10.000 10.000 to 15.000 > 15.000	0 (0,0%) 24 (80,0%) 1 (3,3%) 5 (16,7%)	0 (0,0%) 4 (66,7%) 2 (33,3%) 0 (0,0%)	**0,038**
*Financial services*			
0 to 1.000 1.000 to 2.000 2.000 to 3.000 > 3.000	23 (76,7%) 1 (3,3%) 0 (0,0%) 6 (20,0%)	0 (0,0%) 4 (66,7%) 2 (33,3%) 0 (0,0%)	**< 0,001**
**Variable costs (BRL)**			
*Food kinds*			
0 to 4.000 4.000 to 8.000 8.000 to 12.000 > 12.000	0 (0,0%) 1 (3,3%) 23 (76,7%) 6 (20,0%)	0 (0,0%) 30 (100,0%) 4 (66,7%) 2 (33,3%)	0,717
*Disposable items*			
0 to 400 400 to 800 800 to 1.200 > 1.200	23 (76,7%) 1 (3,3%) 1 (3,3%) 5 (16,7%)	0 (0,0%) 4 (66,7%) 2 (33,3%) 0 (0,0%)	**< 0,001**
*Cleaning products*			
0 to 200 200 to 400 400 to 600 > 600	23 (76,7%) 1 (3,3%) 1 (3,3%) 5 (16,7%)	0 (0,0%) 6 (100,0%) 0 (0,0%) 0 (0,0%)	**< 0,001**
*Extra expenses*			
0 to 350 350 to 700 700 to 1050 > 1050	24 (80,0%) 1 (3,3%) 0 (0,0%) 5 (16,7%)	0 (0,0%) 0 (0,0%) 4 (66,7%) 2 (33,3%)	**< 0,001**
**Net income (BRL)**	20.000	32.500	0,100
	(12.000–20.000)	(18.000-32.500)	
**Profitability (%)**	10 (10–10)	25 (18–25)	**< 0,001**

Note: Values presented as absolute frequency (relative frequency) for categorical variables and as median and interquartile range (25th and 75th percentiles) for quantitative variables

* Mann-Whitney Test for comparing medians and Chi-Square/Fischer’s Exact Test for comparing proportions

The average number of people served in the canteens in the traditional models was greater than 500 students, while in the healthy models it ranged between 300 and 500 students (p < 0.001). In the healthy model canteens, a higher proportion of canteens that took less than a year to recoup their investment was observed (33.0% vs. 0.0%). While in the traditional model canteens there was a higher proportion of canteens that took 1 to 3 years to recoup the investment (96.7% vs. 66.7%). It was found that the profit margin of the most sold product was higher in the traditional model canteens (p < 0.001). In 76.7% of these a profit margin was greater than 100.0%, while in all (100.0%) canteens of the healthy model the profit margin was between 33.0 and 66.0% (Table 3).

Regarding fixed and variable costs, it was observed that among the traditional model canteens there was a greater proportion of the extremes of financial service expenses (76.7% and 20.0% of the traditional model canteens had expenses from 0 to R$1000.00 and > R$3000.00, respectively; while the healthy model canteens had expenses that varied between R$1000.00 and R$3,000.00) and with cleaning (76.7% and 16.7% of the traditional model canteens had expenses from 0 to R$200.00 and > R$600.00, respectively; while the healthy model canteens had expenses between R$200.00 and R$400.00). Healthy model canteens had a higher proportion of infrastructure expenses (66.7% of the healthy model canteens had expenses from 2.500 to 5.000, while 93.3% of the traditional model canteens had expenses from 0 to 2.500, p < 0.001), disposables (66.7% of the healthy model canteens had expenses from 400 to 800, while 76.7% of the traditional model canteens had expenses from 0 to 400, p < 0.001), and extra expenses (66.7% of the healthy model canteens had expenses from 700 to 1050, while 80.0% of the traditional model canteens had expenses from 0 to 350, p < 0.001) compared to traditional model canteens (Table 3). Among the healthy model canteens, higher median profitability was observed compared to the traditional model canteens [25.0% (18–25) vs. 10.0% (10–10), p < 0.001] (Table 3).

Furthermore, no statistically significant differences were observed between the two canteen models for the average number of employees, average initial investment value, gross revenue, total expenses, and net income (p > 0.05).

Considering the managers’ perception about the identification of healthy canteens, all considered it healthy, with two managers of traditional models and all managers of the healthy model classified as “totally healthy”, and two managers of the traditional model classified as “partially healthy”. healthy”. Managers also reported that the socioeconomic profile of students may vary from one school to another, however, the variety of products offered in different schools was similar, with a small change in some cases, generally determined by the contract of each school. Only one canteen manager classified as traditional reported not having tried to include healthier products among the foods and beverages already sold. The added items that were most reported in this case were 50% cocoa chocolate milk, homemade cookies, sweets with fruits or vegetables, fruit, natural sandwich, and natural fruit juice (data not shown).

Regarding the economic viability of healthy canteens, the managers of this model considered it viable, and one of them specified the need to have the snack kit service to increase the company’s revenues. To hire this service, the person responsible for the student signs a contract and when paying a monthly amount, the child receives a snack consisting of a carbohydrate source food (cake, sandwich, savory, biscuit), a drink (natural fruit juice or milk with cocoa or fruit smoothie) and a serving of fruit. As for those responsible for traditional canteens, two believe that the healthy canteen model is unfeasible, and one manager believes that this is due to the worsening economic crisis in the country. Of the managers of traditional canteen models who believe in the economic viability of healthy canteens, one of them justified the need to have the snack kit service in addition to the cafeteria service for profit (data not shown).

## Discussion

In the present study, most of the canteens analyzed were classified as traditional, in which there is a predominance of UPF commercialization. Although half of the managers of traditional canteens believe that healthy canteen models are unfeasible from an economic-financial point of view, the results indicated that in healthy canteens, profitability was higher and the payback time of the initial investment was shorter compared to traditional canteens. The average number of employees, average initial investment value, gross revenue, total expenses, and net income were similar between the canteen models. However, the highest profit margin on the best-selling product and the lowest expenses with infrastructure, disposables, and extra expenses were observed in traditional canteens. To the best of our knowledge, this was the first study to classify and compare the economic viability of traditional and healthy canteens. Thus, it was not possible to compare the findings of this study with the literature concerning traditional and healthy models, and in this sense, it was decided to make a comparison with general data on the profile of foods sold in school cafeterias.

Studies carried out in Brazil show a high frequency of commercialization of unhealthy foods in private school canteens [4–6, 22, 24, 25] evidencing that the traditional canteen model is more frequent in the reality of schools. A study based on a three-year longitudinal dataset from 54 private schools in Brazil, covering purchases made by 20,333 children and adolescents, showed that more than 60.0% of the products offered in private schools have low nutritional value and only 11.0% high nutritional value [7]. Another study carried out in Rio Grande do Sul showed that the most sold foods in canteens were fried snacks and puff pastries, hot dogs, candies, chocolates, and soft drinks [25]. According to data from the PENSE, the products most sold in private canteens were baked snacks, natural fruit juice, and soft drinks [3].

Thus, the presence of canteens that provide more UPF in schools is associated with higher UPF consumption by children and adolescents in this environment [47]. In this sense, there is a growing body of evidence about the impact of UPF consumption on the health conditions of these groups. Studies carried out with children and adolescents have shown that UPF consumption was associated with increased concentrations of total cholesterol [48–51], LDL-c (low density lipoproteins cholesterol) [49, 50], total triglycerides [48, 49], abdominal [51] and body adiposity [52, 53], and dental caries [54], in addition to a decrease in HDL-c (high density lipoproteins cholesterol) [49].

Given this scenario, there is a need to regulate the school food environment to implement healthy canteens in private schools. A study carried out in Brazil showed that adolescents covered by laws restricting the sale of food and beverages in school cafeterias had an 11% lower chance of obesity (adjusted OR = 0.89; 95% CI 0.88–0.91) [55]. In this context, countries such as Australia already evaluate the presence of healthy canteens as an important public health policy, with the school being an ideal place to promote healthy eating [56]. However, there are many barriers to promoting healthy school food environments, such as the lack of involvement of stakeholders (school principals or school food service managers), resistance to change on the part of students, family members, and canteen staff, in addition to stakeholder concern about the profitability, revenue and/or commercial viability of this trade model [28, 29].

However, the results of the present study suggest that the profitability of healthy canteen models is higher than that of the traditional model, with this value being higher than expected for the segment of bars and restaurants (5.0 to 10.0%) [57]. In this perspective, a systematic review [58] showed that of the eight studies with school food services that reported favorable results for health, mainly through a decrease in the sale of unhealthy foods and a simultaneous increase in the sales of healthier products, only one study reported unfavorable commercial viability for this initiative.

In this sense, to ensure the implementation of healthy canteens that are economically viable, it is essential to invest in the training of canteen owners and/or managers [59, 60]. In addition, it is essential to draw up an action plan to plan in an organized and gradual manner the changes necessary for the transformation of the school food environment, considering the cost, feasibility, and execution time of the activities, in addition to having the support and encouragement of all actors involved such as parents/guardians, students, teachers, directors, employees and canteen owners [61]. In this context, documents such as the Food Guide for the Brazilian Population [37], the Healthy Canteens Manual [21], and the Practical Guide to a Healthy Canteen [62] can be used as a reference to help in the process of transforming school canteens into healthier environments.

The results point to the need to establish awareness-raising actions with canteen managers and school directors to deconstruct the idea linked to the low profitability of healthy canteens. In addition, it is necessary to implement national guidelines that guide canteen owners to adapt the marketing of food to promote adequate and healthy food and that consider the local food culture in the school environment, in line with the recommendations of the Food Guide for Brazilian Population [37], and it is essential to monitor the effectiveness of regulation to achieve the proposed objectives [63].

It is also expected that the results of this study can be used to guide public policies aimed at promoting adequate and healthy eating, as well as the prevention and control of obesity and other chronic non-communicable diseases in childhood and adolescence, in addition to supporting actions in favor of a health-promoting school food environment, to contribute to the adequate nutritional status of students and, consequently, to their maximum potential for growth and development.

### Strengths and limitations

The strengths of our study include the novelty in the analysis of the economic viability of canteens in private schools is considered, especially in the model of canteens that sell healthy foods. In this sense, the results address an existing gap in the literature and provide data that can support legislators and decision-makers in the adoption of strategies aimed at promoting a healthy school food environment.

This study has limitations related to carrying out the research remotely and only in the city of Belo Horizonte, which may not reflect the situation of private canteens in other cities and regions of the country; there is also a memory bias due to the data being self-reported by canteen managers. The research focused on descriptive aspects, not having analyzed fiscal documents, accounting, or management reports related to the sale of products, as well as the costs and expenses of the company. In addition, the questionnaire was not validated and a pilot study was not carried out. However, it is emphasized that this instrument was prepared in partnership with specialists in the areas of Administration, Economic Sciences, Accounting Sciences, International Economic Relations, and Controllership and Finance at the Federal University of Minas Gerais. Finally, the food and drinks offered were not checked since only the menu was analyzed, which may not reflect the reality of all products sold in the canteen, the analysis of the list of ingredients of the culinary preparations and of some foods that did not have the brand described on the menu was not carried out, and the presence of nutritionists on the company’s staff was not investigated, which could influence in the availability of food offered in school canteens.

## Conclusions

Healthy school canteens showed better financial and economic results compared to traditional canteens, with emphasis on greater profitability and a shorter recovery time of the initial investment. Traditional canteens demonstrated higher profit margins on the best-selling product and lower infrastructure, disposables, and extra expenses. In the latter model, there was greater commercialization of ultra-processed foods and beverages. The need for actions to clarify the economic and financial feasibility of implementing healthy canteens in school units is reinforced.

## Data Availability

The datasets used and/or analysed during the current study available from the corresponding author on reasonable request.

## Abbreviations

PNAE: National School Feeding Program
PENSE: National School Health Survey
UPF: Ultra-processed foods and beverages
IDHM: Municipal Human Development Index
SEE/MG: Minas Gerais State Department of Education
UCJ: UFMG Consultoria Júnior
UFMG: Federal University of Minas Gerais
DRE: Income statement for the year
SPSS: Statistical Package for the Social Sciences
LDL-c: Low density lipoproteins cholesterol
HDL-c: High density lipoproteins cholesterol
